# Endometritis—Its Pathology, Symptomatology and Treatment*Read before the Medical Association of Georgia, April, 1894.

**Published:** 1894-05

**Authors:** William W. Stewart

**Affiliations:** Columbus, Ga.


### ENDOMETRITIS—ITS PATHOLOGY, SYMPTOMATOLOGY AND TREATMENT.*

By WILLIAM W. STEWART, M.D., COLUMBUS, GA.

In beginning the discussion of this subject, I do so feeling that upon these lines of reasoning hangs the only true conception of endometritis and its treatment; these conclusions are drawn from a large clinical experience and a close study of its pathological conditions. I will classify the conditions as simply as possible, take up its etiology and pathology, and discuss its treatment from the standpoint of its pathology.

We have Simple, Septic and Gonorrheal. Under the head of Simple are gathered all those conditions, inflammatory in character, invading the endometrium, produced by the following causes:

Displacements causing chronic congestion and hypertrophy.

Imprudent exposure during menstruation.

Retention of menstrual flow or mucus by cervical obstruction.

Sexual abuse either in intercourse or masturbation.

Fevers, eruptive and protracted simple.

Frequent miscarriages.

Traumatism by intra-uterine instrumentation or pessaries.

Under the head of Septic are gathered all those causes by which septic material or germs are implanted upon or within the mucosa, as by dirty instruments or hand.

*Read before the Medical Association of Georgia, April, 1894.

Decomposing menstrual blood or lochia infected by lack of cleanliness at menstruation or abortion. (Those forms arising at confinement come rather under the head of puerperal fever.)

Torn perineum, which makes of a normally closed vagina a cavity frequently filled with air, thus opening broad avenues for infection.

Gonorrheal, alone caused by introduction of the gonococci.

We may find the pathology of simple metretis one of two forms: Primarily, and by far the most common, is a hypertrophied condition of the mucous membrane, folicles tortuous, but retaining in its integrity its epithelial covering, which presents a swollen appearance; muscular walls thickened, often causing decided enlargement of the uterine body; blood vessels engorged and turgid, aiding in the local œdema; small capillaries multiplied in number and distended with blood, bleeding freely on slight traumatism or over exertion; crypts between folicles filled with glary mucuus and lymphoid cells, which are constantly discharged as a leucorrheal discharge.

From the supernutrition produced by the over supply of blood, there often springs into existence fungosities, which are truly hyperplastic cells, or following a confinement or abortion, small particles of decidua or placenta may remain which take on new life and growth, forming fungoid masses. In either condition there is constantly thrown off a mucoid discharge, varying in color by the number of lymphoid and desquemated epithelial cells which it contains. Either of these growths at menstrual period may produce metrorrhagia and often alarming hemorrhage.

Secondly: The atrophic form in which the uterine walls are hardened by schlerosis, the mucous membrane almost devoid of epithelium and closely adherent to submucosa, crypts few in number. This condition is always a secondary one, and is invariably associated with amenorrhea and dysmenorrhea of a distressing and intractable form.

The symptoms and many complications arising from endometritis are of much interest, illustrating more perfectly the effect of the sympathetic nervous system on the different organs of the body and the general health than any other disease. The leading symptoms of endometritis are headache, occipital and parietal in location, flatulence, indigestion, fermentative in character, produced by dilation of the stomach, which is almost constantly associated with endometritis of long standing, pain in either breast, usually the left, great fatigue from exercise, leucorrhea distressing in character, in the acute forms quite profuse and sometimes of very bad odor. In the atrophic form the leucorrhea is absent or very slight, but all other symptoms are usually present in aggravated form. In both forms, the nervous system is thoroughly shattered, often producing hysterical phenomenon whose form is legion. Sufferers from this trouble quickly develop a decided cachexia, often suggestive of malignant trouble though none exists.

The treatment of the simple acute endometritis is almost sure to yield prompt and good results when the condition does not exist in an aggravated form, and can be taken early. The greatest benefit and flattering results can be obtained by change of air, well regulated hygiene, exercise and nutritious diet, combined with a judicious administration of chalybeates and arsenic. At menstrual period rest in bed throughout its course, to be continued for at least four successive months. Metrorrhagia and dysmenorrhea, if they exist, are well controlled by the judicious use of fluid extract of vibernum prunifolium, hydrastis and canabis indica.

It is, however, the misfortune of both physician and patient, that when the metritis first makes its appearance, it is looked upon as a matter of small moment, the result only of a bad cold or over-exertion ; or, if the family physician is called, he is too prone to pass it by unheeded, with the remark : "If it troubles you, let me know." By slow degrees, the once simple condition passes into one which demands attention. And let me urge that that attention be prompt and decisive ; for only by such measures will perfect results accrue. There are three lines of treatment to be selected from under these conditions, depending upon the variety and extent to which the process has advanced. In the simple acute, just passing into the more aggravated forms, and in the atrophic variety, simple gauze packing will in many instances prove all that is required.

To insure good results in performing this operation and to steer clear of the rock of sepsis, upon which so many surgical crafts are wrecked, it must be borne in mind that the vagina contains at all times thousands of pyogenic cocci, and it would be easy through this medium to infect a previously uninfected endometrium, thereby exchanging the simple for the septic form, with its legion of complications.

To avoid such dire results, the strictest laws of antisepsis should be observed. A vaginal douche of one to two thousand sublimate solution should be given; then the external genitals should be thoroughly scrubbed with tincture of green soap and warm water, a perfectly clean speculum then introduced and vagina and cervix well wiped off with pledgets of absorbent cotton, dipped in a sublimate solution of one to two thousand; cervix should then be caught with a tenaculum and drawn forward, thereby straightening cervical canal; cervical canal should then be wiped out with absorbent cotton on an applicator having previously been emerged in same solution. If necessary, cervical canal should then be slightly dilated, cervix being steadied by tenaculum, a long strip of iodoform gauze three-quarters to one inch in width, and from fifteen to twenty per cent. in strength is looped over the end of a smooth uterine sound, and gently introduced to fundus; the sound is then withdrawn and point again entangled in gauze at the mouth of cervical canal; and gently introduced, thus loosely packing the uterine cavity with gauze. It is then clipped off about one inch from cervix, and a wider strip is introduced in the vagina over and around cervix. Covering this with a pledget of sterilized or borated cotton, and operation is then complete.

This dressing is allowed to remain unmolested four or five days, patient preferably remaining in bed. This, however, is not absolutely essential to a successful result, as she may be permitted to remain upon a sofa or recline in an easy chair.

On the fourth or fifth day this dressing is removed, with the same precaution, and again packed. This procedure, is repeated if necessary, four or five times. If menstrual flow should appear, remove dressing and repack fourth day after its disappearance.

This treatment insures drainage and an aseptic healing dressing to an inflamed and œdematus mucous membrane in the more acute forms, and to a mucous membrane below the normal in its nutrition in the atrophic. In both instances, a perfectly scientific procedure which recommends itself from every standpoint of common sense and good surgery.

In the acuter forms, this treatment, by drainage and antiseptic dressing, quickly brings about resolution, and restores the engorged uterus to its normal condition. In the atrophic, it stimulates and insures better nourishment to an impaired and partially destroyed mucous membrane, and thereby often, to some extent, removing the troublesome dysmenorrhea and amenorrhea.

In the severer forms of the septic, which have become chronic in character, with troublesome symptoms, curettage should precede the gauze packing as already described.

The septic form in its acute invasion, presents a picture, once seen, never to be forgotten, and as differentiation between gonorrheal and septic can only be made by the microscope, this clinical picture will represent both:

A patient, after miscarriage, abortion dirty instrumentation, the wearing of a stem pessary or the introduction of the gonococci is first taken with headache and languor, followed by chills and fever, usually high, micturition is usually frequent, urine small in quantity, bowels constipated, tympanitis more or less constant, depending upon extent of invasion of pelvic peritoneum, pelvic region tender on pressure, and cholicy uterine pains gradually becoming a dead ache. Examination reveals vagina hot and bathed in mucopus, more or less hemorrhagic, uterus engorged and exquisitely tender to touch, exudative masses may be found surrounding uterus if adnexia be involved.

By speculum examination a large, turgid, often bluish cervix is xposed, from which there is constantly discharged a ropy mucopus; cervix may be eroded, and in cases of late abortion and post partum origin, patches of diphtheritic membrane may be found upon cervix and vaginal walls, due to the presence of the streptococci.

Endometritis, when produced by the gonococcus, is usually less rapid, and rarely causes death; whereas, in the septic form, death not infrequently draws a veil around the victim.

Whenever these conditions present themselves, never hesitate; act with prompt, thorough, clean and determined intent; with such, good results will surely come, and many lives of suffering and invalidism caused by diseased adnexia will be saved. Treat the disease as common sense and good surgical rules surely dictate.

What surgeon is there to-day, who, having such a condition in any other portion of the body, would not make it his first step, his foundation for further treatment, to thoroughly cleanse the septic cavity, following it by good drainage.

Strike off the shackles of ignorant timidity, which holds to vaginal douches, hot poultices and leads to high mortality.

To thoroughly cleanse this cavity, by referring to its pathology, it is found that staphylococci and streptococci are almost entirely found in the mucosa, rarely penetrating to the muscular wall; mucous membrane is bathed with pus, and in aggravated forms the mucosa at times necrotic.

In the gonorrheal, the gonococci rarely pass deeper than the lymph spaces beneath the epithelial lining.

This being clear, the first step suggested is to remove the infected tissue, which should be done thoroughly; a half procedure is worse than none. This should be procured by curettage, a description of the method of which is given later.

The presence of pelvic exudate is no contra-indication, but rather an indication for curettage.

Curettage, under all circumstances, should be performed with the greatest care. Bowels should be thoroughly evacuated the night prior to operation, with free doses of salts; one hour prior to administration of anesthetic, an enema of warm water should be given; vaginal douche of one to two thousand sublimate solution should then be given, and external genitals should be well scrubbed with hot water and tincture of green soap, after which the vulva should be covered with a pad of sublimate gauze held in position by napkin.

After anesthesia is complete, instruments being sterilized, a speculum should be introduced, and any mucus that may have collected in vagina washed out with stream of sublimate solution one to three thousand; vaginal walls and cervix should then be washed with pledgets of absorbent cotton soaked in same solution, and cervical canal cleansed with cotton wrapped upon an applicator, immersed in either carbolic acid, pure, or sublimate solution; cervix should then be caught by tenaculum, drawn down and steadied, dilator introduced and cervical canal well dilated; intra-uterine washer should then be introduced, and uterine cavity washed with sublimate solution one to six thousand, after which dull curette is introduced to locate any decidual tufts, polypi or granulating masses; sharp curette should then be used with slow, regular strokes from fundus to external os; mucous membrane should be stripped off thoroughly, when sides have been completed, fundus should be treated in same manner, and successively curette should be rotated in each cornua; curette should continue till the sensation peculiar to the contact of the curette to muscular tissue is detected, and in all cases where uterus is not intensely softened, as in the puerperal state, there is a sound produced, harsh and grating in character, when the muscular wall is encroached upon by the curette; if, as in the puerperal uterus, the muscular wall is very soft and danger of perforation is great, a rather flat, broad and moderately sharp curette should be used, and great care should be exerted to detect immediately the sensation produced by the muscular coat upon the curette.

Though the danger of perforation by sharp curette is so loudly sung, in careful hands, skilled in such work, such an accident should never occur. After curettage, uterus should again be washed out with sublimate solution, one to six thousand, till water returns clear; vagina should then be thoroughly cleansed, and uturus well packed, as already described. Vagina should be loosely filled with strips of gauze, and vulva pad of sublimate gauze applied, to be changed every six hours; water should be drawn at each dressing, and dressings allowed to remain unmolested as long as temperature remains down, till four days shall have elapsed, when it should be removed as before. If temperature remains up, which is unlikely, dressings should be removed after twelve or twenty-four hours, as indicated by temperature, and uterine cavity again douched with sublimate solution one to six thousand, and uterus repacked as before. If temperature remains down, dressings can be left from five to six days, when they should be removed with antiseptic precaution; this treatment being continued till recovery is complete; the gauze insures perfect drainage, and with this plan of treatment laid down, temperature quickly falls, and pelvic exudations soon disappear.

In the septic and gonorrheal forms, peritonitis may exist, and if so, bowels should be flushed daily by salts; should nausea exist, precluding the use of salts, an enema of

| | |
|---|---|
| Glycerine | oz. 6. |
| Oxgall (fresh) | oz. 1. |
| Rochelle salts | oz 6. |
| Aquæ qs | qts. 1. |

M. Use as one enema.

This should be given thirty inches up the bowels by high enema tube, and repeated as required.

By this treatment, rest and comfort far greater than by morphine will be produced, and "nature's peritoneal drainage tube," as Lawson Tait has termed the intestinal canal, is constantly relieving the peritoneal cavity and general system of noxious material.

Beware of the delusion, often by temerity becoming a snare, of making applications of caustic agents to cervical canal as there exist authority for many writers.

The best result that can be hoped for by such treatment is temporary amelioration of the condition; if the simpler forms are not made septic by the absorption of necrotic tissue produced by these agents, it goes on from a simple congestion, thoroughly curable, to the atrophic form, virtually incurable.

Caustics destroy the mucosa, and in its place scar tissue is substituted, through which the patient is compelled to menstruate. The sufferings of such a one may well be imagined.

In the cervical canal the severe caustics lead to cystic degeneration of cervix, produced by cicatricial contraction closing the ducts of the cervical glands.

On the other hand, after curettage and gauze packing, the mucosa is regenerated fresh and healthy.

